# Development and In-Silico and Ex-Vivo Validation of a Software for a Semi-Automated Segmentation of the Round Window Niche to Design a Patient Specific Implant to Treat Inner Ear Disorders

**DOI:** 10.3390/jimaging9020051

**Published:** 2023-02-20

**Authors:** Farnaz Matin-Mann, Ziwen Gao, Chunjiang Wei, Felix Repp, Eralp-Niyazi Artukarslan, Samuel John, Dorian Alcacer Labrador, Thomas Lenarz, Verena Scheper

**Affiliations:** 1 Lower Saxony Center for Biomedical Engineering, Implant Research and Development (NIFE), Department of Otorhinolaryngology, Head and Neck Surgery, Hannover Medical School, Stadtfelddamm 34, 30625 Hannover, Germany; 2 Cluster of Excellence “Hearing4all” EXC 1077/1, 30625 Hanover, Germany; 3 Ear Nose and Throat Institute and Department of Otorhinolaryngology, Eye & ENT Hospital, Fudan University, Shanghai 200031, China; 4 OtoJig GmbH, Karl-Wiechert-Allee 3, 30625 Hanover, Germany; 5 HörSys GmbH, Karl-Wiechert-Allee 3, 30625 Hannover, Germany

**Keywords:** inner ear disease, medical image segmentation, individualized implant, additive manufacturing, round window niche, round window membrane

## Abstract

The aim of this study was to develop and validate a semi-automated segmentation approach that identifies the round window niche (RWN) and round window membrane (RWM) for use in the development of patient individualized round window niche implants (RNI) to treat inner ear disorders. Twenty cone beam computed tomography (CBCT) datasets of unilateral temporal bones of patients were included in the study. Defined anatomical landmarks such as the RWM were used to develop a customized 3D Slicer™ plugin for semi-automated segmentation of the RWN. Two otolaryngologists (User 1 and User 2) segmented the datasets manually and semi-automatically using the developed software. Both methods were compared in-silico regarding the resulting RWM area and RWN volume. Finally, the developed software was validated ex-vivo in N = 3 body donor implantation tests with additively manufactured RNI. The independently segmented temporal bones of the different Users showed a strong consistency in the volume of the RWN and the area of the RWM. The volume of the semi-automated RWN segmentations were 48 ± 11% smaller on average than the manual segmentations and the area of the RWM of the semi-automated segmentations was 21 ± 17% smaller on average than the manual segmentation. All additively manufactured implants, based on the semi-automated segmentation method could be implanted successfully in a pressure-tight fit into the RWN. The implants based on the manual segmentations failed to fit into the RWN and this suggests that the larger manual segmentations were over-segmentations. This study presents a semi-automated approach for segmenting the RWN and RWM in temporal bone CBCT scans that is efficient, fast, accurate, and not dependent on trained users. In addition, the manual segmentation, often positioned as the gold-standard, actually failed to pass the implantation validation.

## 1. Introduction

The incidence of inner ear disorders—e.g., idiopathic sudden sensorineural hearing loss (ISSHL) and Meniere’s disease (MD)—in the population of industrialized countries, is estimated at 5–20 per 100,000 people annually for ISSHL and 513 per 100,000 people annually for MD [1,2,3,4,5,6]. ISSHL is defined as sensorineural hearing loss of more than 30 dB in over three consecutive frequencies in less than three days [1,7]. Meniere’s disease (MD) is an idiopathic inner ear disorder characterized by spontaneous attacks of vertigo, fluctuating sensorineural hearing loss especially, in the low frequencies, tinnitus, and aural fullness [8,9,10]. The modern therapy for these inner ear disorders is increasingly shifting to local drug delivery to the inner ear. Systemic pharmacotherapy (oral or intravenous) needs the application of high drug concentrations to receive biologically relevant drug levels in the ear, always being in danger of massive side effects. Local drug delivery reduces the drug amount needed and is achieved via middle ear application from where the drug is intended to diffuse into the inner ear. But obtaining a high concentration of the drug in the inner ear over a period of weeks is a challenging issue for any drug administered into the middle ear cavity via needle injection through the tympanic membrane [11,12].

**Figure 1:**
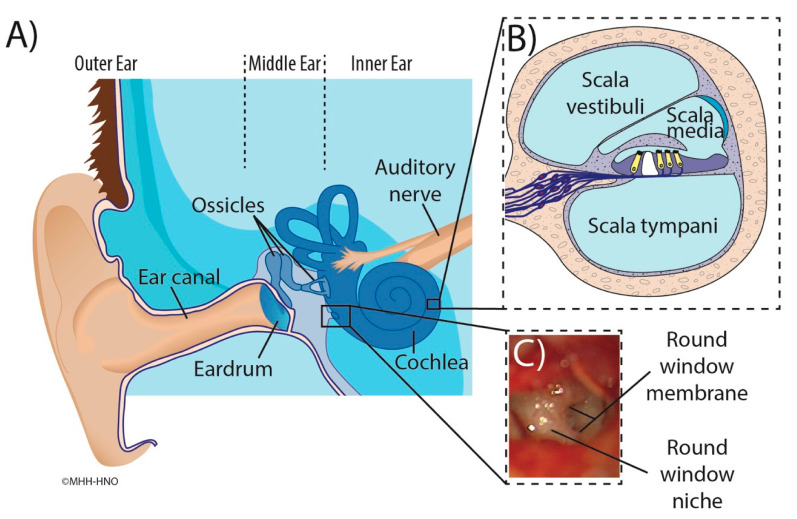
Illustration of the human ear anatomy. Main figure (**A**): Overview of the outer ear, ear canal and structures of the middle ear containing the tympanic membrane (eardrum), round window niche, and ossicles. The cochlea and auditory nerve are also shown. Inset (**B**): Cross-section of the cochlea illustrating the three fluids filled compartments scala vestibuli, scala media with sensory cells (yellow), and scala tympani. Inset (**C**): Intraoperative microscopic appearance of the round window region seen through facial recess with focus on the round window niche and round window membrane [15].

The temporal bone is a major part of the lateral skull base that contains critical structures including the middle ear, the inner ear, cranial nerves, and numerous vessels [13]. The only two connections between the middle and inner ear are the oval and round windows that are covered by the stapes footplate and round window membrane (RWM) [14], respectively. The RWM is a semi-permeable membrane that, in human temporal bones, is located deep in a recess called a round window niche (RWN) that is formed by individually very differently shaped bones (Figure 1) [15,16,17,18,19].

To achieve a sustained drug delivery to the inner ear, the substance has to be supplied continuously at the RWM resulting in continued diffusion to the inner ear. Thereby, a high concentration of active ingredient would be achieved locally, while the systemic burden on the organism remains low. Side effects for the patient can be significantly reduced. A new approach that offers the potential for sustained inner ear local drug delivery is an additively manufactured, patient individualized, drug-loaded implant that fits precisely into the RWN. In order to manufacture such an individualized implant, a three-dimensional (3D) representation of the patient specific RWN is constructed based on image segmentation of a computed tomography (CT) or cone beam computed tomography (CBCT) scan of the temporal bone. Manual segmentation—manual slice-by-slice identification and outlining of the relevant anatomy of the RWN in CBCT scans of temporal bones using a computer software—is time consuming and requires considerable effort by trained technicians or clinicians [20,21]. Consequently, the manual segmentation blocks a lot of working time of a highly qualified employee, which may only be implemented in a few individual cases in the clinic, but not for routine implementation. This is true for preoperative manual segmentation of the structure of interest, i.e., the RWN, and especially in the case of intraoperative manufacturing of implants. An automated algorithm that identifies structures within the temporal bone anatomy, being highly accurate and requiring only little input from the otolaryngologist could speed up the 3D segmentation of the RWN considerably and removes, in parallel, bias from the manual process [22].

Current segmentation approaches of medical images represent the structures of interest by identifying image voxels based on their intensity level variations, or Hounsfield values (HV) [13].

**Figure 2:**
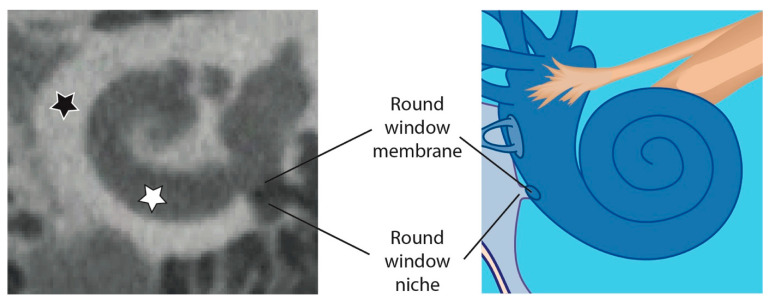
Overview of the human cochlea in a CBCT image (**left**) compared to an illustration of the cochlea (**right**). The white star marks the fluid filled cochlea which is surrounded by radio dense hard bone (black star). The RWN is also surrounded by hard bone and the volume of the RWN is mainly air filled (as shown in the CBCT image on the left) but can also be fluid filled or obstructed by tissue.

The process of auto-segmentation of the inner ear is facilitated by the fact that the cochlea is a fluid filled structure mainly surrounded by radio dense hard bone (Figure 2), providing consistent contrast against its surroundings [23]. In the temporal bone anatomy mainly three HV have to be differentiated in the process of RWN segmentation: the bony structures surrounding the RWN have high HV, the middle ear which is air filled has low HV and the HV of the fluid filled cochlea are in between (Figure 2). As shown in our previous work, the identification of the RWN and surroundings is feasible but the manual segmentation is very time-consuming [15]. In some temporal bones the identification of the RWN volume in CBCT scans may prove difficult because anatomic obstructions that can block the RWM, such as adhesions, postoperatively aroused scar tissue, the false membrane, or thickened membranes [24], can result in similar HV as the RWM or cochlea.

To date, several software tools that can enhance and accelerate the segmentation of structures in the temporal bone have been developed [25] but none of them have focused on segmentation of the RWN volume.

In our prior study an otolaryngologist used her anatomical knowledge in addition to the image intensity and manually segmented the anatomy of 50 RWN and found variations in volume and shape of the RWM and RWN [15]. However, using the same software tool for developing and subsequent clinical transfer of novel round window niche implants (RNI) is impractical due to the labor-intensive step of manual segmentation of imaging data and requires highly trained specialists for identification of the RWN anatomy. Therefore, our overall goal was to develop a semi-automated segmentation approach that identifies the RWN and the critical surface structures of the RWM for use in the development of patient individualized RNI. To achieve this in an acceptable time, we used an adaptable model of the cochlea that includes a RWM and controls to describe the extent of the RWN complementing a thresholding based segmentation of the bony structures.

To verify the accuracy of the developed semi-automated approach a comparison of 20 clinical cone beam computed tomography datasets of unilateral temporal bones was performed by semi-automated segmentation using a customized 3D Slicer™ plugin with a previous manual segmentation of these datasets.

The applicability of the developed software was verified in three body donor implantation tests. The respective region of interest (ROI) was imaged, the developed semi-automated segmentation approach was used to generate a RWN reconstruction, a RNI was built by additive manufacturing and the implantation feasibility and fitting accuracy were evaluated in the respective donor and compared to a RNI made for the same RWN based on manual segmentation.

## 2. Material and Methods

In order to develop an individualized RNI we wrote a software tool that assists the user to create a suitable 3D model that is based on a CBCT volume image. The software was validated by additively manufacturing RNIs and performing implantation tests to determine implantability and therefore the suitability of the software for clinical use. To assess the time saved for the users, we compare the developed software to the manual segmentation procedure.

### 2.1. Image Acquisition

Twenty anonymized unilateral temporal bones CBCT datasets of patients were included in the study. The protocol for this retrospective study for using the patient’s data was approved by the responsible ethics committee approved (Project identification code 3699-2017). The selected patients were included based on no history of oto-surgical manipulation, no diseased or malformed cochleae. A clinical 3D ACCUITOMO 170 Digital CBCT scanner (J. Morita Tokyo mfg. Corp., Tokyo, Japan) was used for scanning the patients. Resulting CBCT volumes with an exposure time of 30.8 s and a computed tomography dose index of 8.04 mGy and were reconstructed in an isometric voxel size of 0.08 mm × 0.08 mm × 0.08 mm and exported as Digital Imaging and Communications in Medicine (DICOM) data using i-Dixel software version 1.68 (J. Morita Tokyo Mfg. Corp., Tokyo, Japan) [15]. The semi-automated algorithm works independently of different image resolutions and even with non-isometric voxel sizes. However, we recommend a resolution of at least 0.3 mm × 0.3 mm × 0.3 mm.

Like in our earlier work [15], for each dataset, we manually fitted a model of the cochlea using three points (apex, basal turn, round window center) allowing us to define positions and directions in relation to the cochlea using the cochlear coordinate system (CCS) [26]. The CCS is defined as z-axis along the modiolus axis pointing in the direction of the helicotrema, and the x-axis pointing to the RWM. For aligning the RWN segmentations, we shift the origin of the CCS to the RWM-center. This CCS is used to initially place the four control points for the RWM based on a mean-model from an earlier work with µCT data and also to set the ROI for the thresholding of the bone.

### 2.2. Software

As depicted in Figure 2 the RWN is the indentation of the cochlea promontory that is limited by the RWM and is open towards the middle ear.

**Figure 3:**
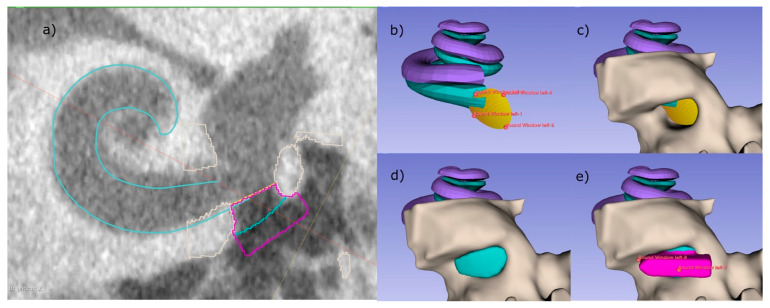
RNI creation using the developed semi-automated software as 3D Slicer™ plugin. (**a**) A CBCT slice of the basal turn of the scala tympani (cyan) with the implant including handle (pink) sitting inside the RWN. (**b**–**e**) 3D visualization of the individual steps, that are also visible as outlines in (**a**). (**b**) Four control points are placed, defining the RWM (yellow) and limiting the backside of the implant body. (**c**) The bone (light gray) surrounding of the RWN is segmented by thresholding, allowing the RWN to be filled (cyan, **d**). In a last step, a handle is added to the implant (pink), helping to define the orientation of the implant.

In the following paragraphs, each step within the development of the software is described in detail. In short, the boundary between the inner ear and the middle ear, i.e., the RWM, was defined by applying an oval cut-out of a saddle shaped surface with four control points (step 1). The bony area was identified by thresholding the CBCT intensities within the ROI (step 2). After the user determines the boundary of the RWN towards the middle ear, the RWN, which will be filled by the implant-body, is completely defined (step 3). The user can add and adjust a handle to the implant that can be used by the surgeon to hold the implant with forceps (step 4; Figure 3e). Since these steps, are performed by labelling voxels, the implant model is converted to a surface model (STL file) using 3D Slicer™’s build-in tools.

To perform these steps, a cubic ROI with an edge length of 5 mm, centered at the estimated position of the RWN is cropped from the CBCT scan (Figure 3c). When using voxel sizes bigger than 0.1 mm × 0.1 mm × 0.1 mm, the software internally up samples the cropped volume in order to segment and create a smooth model of the implant. For each voxel, coordinates are determined in order to relate them to the boundaries defined in step 1, 3 as well as the handle dimensions from step 4.

1. The round window membrane cannot be identified in clinical CBCT images. At best, in really good images, it is possible to identify a slight contrast between the air in the RWN and the liquid in the cochlea to determine the boundary between middle and inner ear. To overcome this, a saddle shaped surface model of the RWM, which is represented by a bilinear interpolation of four points, is implemented (see Figure 3b) and allows to determine if voxels are inside or outside the cochlea.

The initial location of these four points relative to the center of the RWM is based on a mean position that has previously been determined in high resolution µCT scans. In µCT datasets of temporal bone specimens that were scanned with a voxel size of 16 µm × 16 µm × 16 µm, we placed four fiducials in a way that the bilinear interpolation fitted the anatomical structure to a high degree. The mean positions were obtained by repeating this for six RWMs and averaging the positions within our CCS. The user can place the mean RWM model in the clinical scan and manipulate the points in 3D in order to fit the structure of the individual RWM and cochlea.

**Figure 4:**
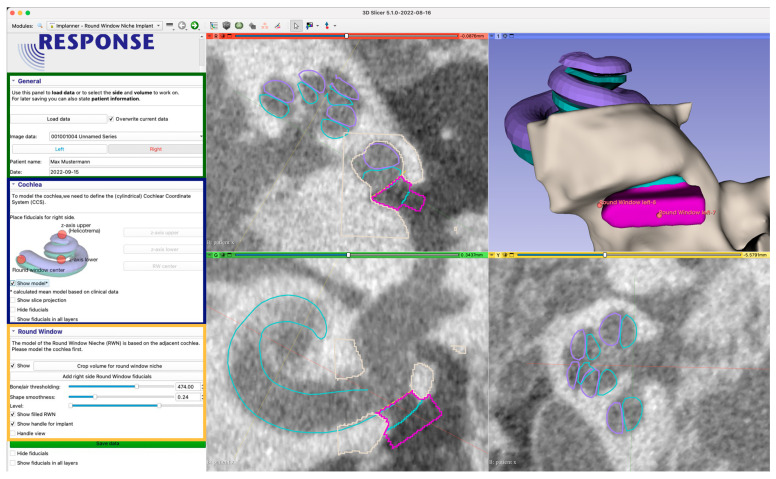
User interface of the developed 3D Slicer™ plugin. In the left side, the user is guided through the process (zoomed in image of the user interface on the left). The green section contains information for data management (patient id, date) and the working orientation (left/right). The blue section is used to place three initial fiducials (control points), for fitting the mean cochlea model. The third yellow section enables the user to add and adjust the adjacent RWN fiducials and to shape the resulting model. The dialog is also used to add a handle and to finally export the data for 3D printing. The right side shows three orthogonal image planes as well as a 3D-rendering of the segmented structures (beige/gray) and derived models. The views are used to interact with the medical image by achieving the accurate placement of control points. By placing the first three fiducials for the z-axis upper and lower points and the RW-center, a mean cochlea model consisting of the scala tympani (cyan) and the scala vestibuli (violet) is placed. The view also shows the model filling the RWN (also cyan) and the handle (pink) with additional fiducials to move and scale the handle.

2. To threshold the bone around the RWN, fitting the peaks for bone, soft tissue, and air in the intensity-histogram of the ROI provides an initial estimation for the threshold value for bone. This value can further be refined by the user. In case that the CBCT image has a high noise level, a slight Gaussian blurring with a kernel width typically in the order of one voxel can be applied by the user to obtain a smooth surface of the implant.

3. Since the RWN is a half-open structure without a clear border towards the middle ear cavity, the extend towards the middle ear is somewhat arbitrary. The user can refine the extent to which the niche is filled by dragging a slider in the user interface (UI) (Figure 4). To understand the effect, it can be useful to think of the niche as a crater lake where the bottom of the lake is the RWM. The shape of the bone around the round window niche provides the topology of the crater, the border of the niche towards the middle ear limiting the maximal water level at which it would spill over the lowest part of the crater border.

**Figure 5:**
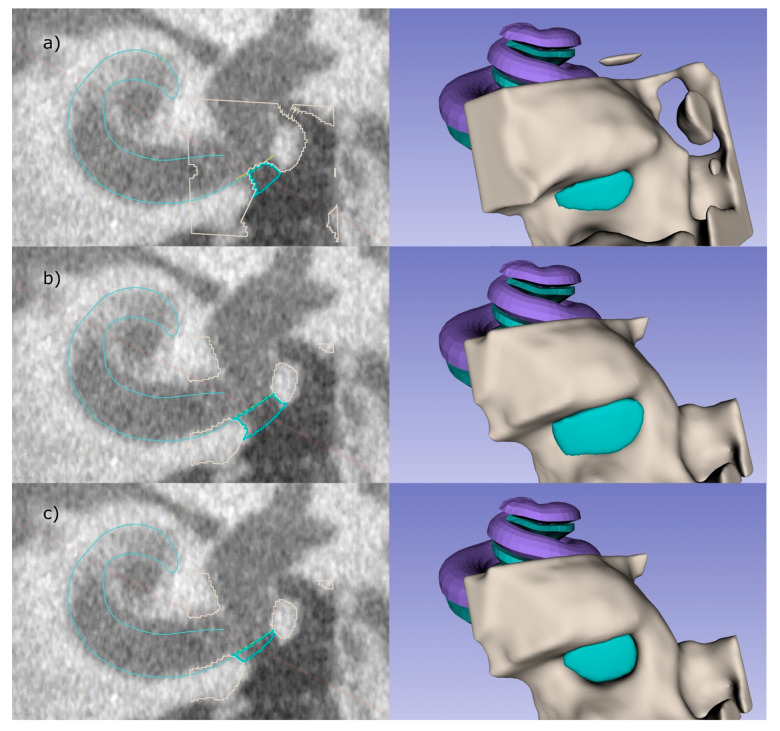
The user has several options to influence the final shape of the implant: (**a**) shows the lower threshold for bone, resulting in a smaller implant compared to the automatic threshold used in (**b**). In (**c**) the level to which the niche is filled is manually reduced. The right images illustrate the RWN-filling corresponding to the related left images.

The user can not only influence the “water level”, i.e., the RWN filling, interactively in the UI (Figure 5) but also the center of gravity by dragging a control point in 3D. Calculating the distance of each voxel to this center allows to exclude voxels above the “water level” therefore completely defining the shape of the niche.

4. A handle for the implant is created on the surface of the implant facing the middle ear (Figure 3e). The shape of the handle was chosen to be a cuboid with one side being pointy in order to provide information about the orientation of the implant. By default, this side is facing the direction of the basal turn of the cochlea at the round window, helping the surgeon to find the supposed fit in the niche. The dimension of the handle can be adjusted by the user, while the software takes care that there is no overlap with bony structures of the middle ear, based on the provided threshold.

### 2.3. In-Silico Validation: Semi-Automated vs. Manual Segmentation

Manual segmentation: An experienced otolaryngologist, highly specialized in segmentation of the temporal bone, performed the manual segmentation of the 20 CBCT datasets using 3D Slicer™ version 4.11 (http://www.slicer.org, accessed on 12 January 2022) (Surgical Planning Laboratory, Brigham and Women’s Hospital, Harvard Medical School, Boston, MA, USA) [27]. The RWN was manually segmented slice wise using a threshold supported paint segmentation technique as described in detail in our previous study [15]. In short, four points were placed to define the cochlea and the RWN: one at the midmodiolar apex, one at the midmodiolar basal turn, one at any point of the RWN and the last was set on the bony tip of the RWN. Then the RWN volume was manually segmented in each slicing plane of the datasets [15].

Semi-automated segmentation: Two otolaryngologists, one experienced and one at the beginning of her residency, performed individually, after a brief explanation of the new software, the semi-automated segmentation as described above on each of the 20 CBCT scans.

Data analysis: Aiming to compare the segmentation methods, we focus on the volume of the RWN calculated by counting the voxels of the implant, before adding the handle and multiplying it with the voxel volume. We calculated the Dice similarity coefficients (DSC) and Jaccard indices (J). In order to better understand where the differences between the manual and semi-automated segmentation arise from, we remove voxels from the manual segmentations that would not be classified as implant by applying the steps 1–3 described in the text above:

Step (1) Removing voxels inside the RWN model.
Step (2) Removing voxels classified as bone.
Step (3) Removing voxels that are above the “spill-over” filling level.

In addition, we compared the area of the RWM calculated based on the number of voxels making up the contact surface between niche and scala tympani.

The results of the semi-automated segmentation were compared to the manual segmentation of the same 20 CBCT scans.

### 2.4. Ex-Vivo Validation: Blinded, Comparative RNI Implantations in Human Cadaver Temporal Bones

To evaluate the implantation feasibility and fitting accuracy of RNI generated from manual segmentations in comparison to RNI generated from the semi-automated segmentation, three previously anonymized formalin-fixed human temporal bones—with in total three RWN—were implanted. The use of human temporal bones was approved by the responsible ethical committee and registered under the number 9236_BO_K_2020. An experienced otorhinolaryngologist performed all manual and semi-automated segmentations of the RNI as described above and implanted and compared the 3D printed individualized RNI (see below). The implantation trials were blinded as the otorhinolaryngologist did not know which RNI—manually segmented or semi-automatically segmented RNI- were handed for the corresponding temporal bone to avoid bias.

For the development of the corresponding RNI, a mobile intraoperative CBCT scanner (xCAT ENT portable CBCT scanner (Xoran Technologies LLC., Ann Arbor, MI, USA)) was utilized for image acquisition. All images were captured with an isometric voxel size of 0.3 mm × 0.3 mm × 0.3 mm. All the segmentations of these CBCT images were done by the same experienced otorhinolaryngologist who performed the manual and semi-automated segmentations of the 20 temporal bones mentioned above.

After the RNI were printed (see the description of the printing process below) the fitting accuracy of the manually segmented and semi-automatically segmented RNI was evaluated and compared based on surgical visual judgment and tactile feedback regarding correct representation of the surgical approach, the general handling of RNI with the handle and the fitting accuracy of the RNI. The insertion was done by an experienced otorhinolaryngology surgeon (User 1) and a conventional transmeatal approach through the external ear canal was performed to visually assess the RWN using a surgical microscope (OPMI PROergo S7 (Carl Zeiss Meditec, Jena, Germany)). For the insertion of the RNI standard surgical forceps were used.

### 2.5. Additively Manufactured Individualized Round Window Niche Implants

After the manual and semi-automated segmentation, a Standard Tesselation Language (STL) file of each digital model was generated as a routine function in 3D Slicer™ and used for 3D printing. The STL file was loaded into the Perfactory RP software version 3.2.3594 (EnvisionTEC GmbH, Gladbeck, Germany) and was sliced into 160 μm slices (80% of the needle diameter, detailed below). The resulting file was imported to EnvisionTEC Visual Machines software version 2.10.130r12, where the model was assigned an infill with a fiber spacing of 0.2 mm and a 90° layer-to-layer rotation, and a single contour outline. The RNI were 3D printed using a 3D-Bioplotter^®^ Manufacturers Series (EnvisionTEC GmbH, Gladbeck, Germany), equipped with a low temperature printing head operated by pneumatic pressures of 5 bar and an UV Curing Head at 365 nm. Medical grade UV silicone (60A MG, BIO-83-6001, Momentive Performance Materials inc., Waterford, NY, USA, silicone elastomer curing at 365 nm) with its silicone catalyst (catalyst compound, Momentive Performance Materials inc., Waterford, NY, USA) in a ratio of 50:1 was prepared using the Speedmixer*™* DAC 150.1 FVZ (Hausschild & Co. KG, Hamm, Germany) for two minutes operated by 3500 rpm. The silicone was loaded into the low temperature head attached with a 200 μm dispensing needle tip (Nordson Australia Pty Ltd., Sydney, Australia) and printed at 27 °C at a movement speed of 2 mm/s. The silicone was crosslinked layer-by-layer using the UV-light head of the printer.

## 3. Results

**Figure 6:**
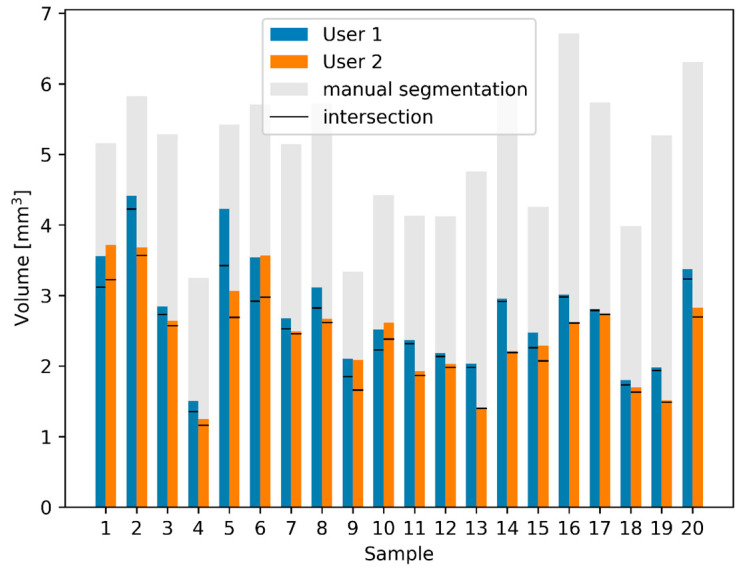
Comparison of calculated RWN volumes and intersections between manual and semi-automated segmentations. The blue and orange bars show the volume of the semi-automated segmentations as performed by the two users. Using the semi-automated method, only small differences between the two users exist. The manual segmentations have a much larger volume (gray). The black lines in each bar represent the volume of the intersection between manual and semi-automatic segmentations.

Comparing the volume of the RWN of the semi-automated segmentation of the two users with the manual segmentation (Figure 6), one can see that the results of the 20 semi-automated segmentations of User 1 and User 2 are a lot more similar than the manual segmentation. The volume of the RWN semi-automated segmentations of User 1 was 13 ± 12% smaller than the volume of the RWN segmentation of User 2. The area of the RWM of the semi-automated segmentations of User 1 was 5 ± 17% smaller than the area of the semi-automated RWM segmentation of User 2. While there were similar results of the semi-automated segmentations of User 1 and User 2, we only compared the results of User 1 to the manual segmentation and describe the results in detail below.

### 3.1. In-Silico Validation: Semi-Automated vs. Manual Segmentation

The manual segmentation for labeling the RWN took around 30 min for each unilateral temporal bone dataset, whereas using the semi-automated application took only three to five minutes and that included the creation of the RNI STL file for 3D printing.

The bars of the manual and automated segmentations show a clear correlation (Pearson correlation coefficient [28]: r = 0.62, *p* = 1.7 × 10^−5^), the volume of the semi-automated RWN segmentations were 48 ± 11% smaller than the manual segmentations. This difference in volume between semi-automated and manual segmentation is quantified by calculating the DSC the Jaccard index as shown in Table 1.

The area of the RWM of the semi-automated segmentations was 21 ± 16 % smaller than the manual segmentation.

While the difference might be explained by the fact that the manual segmentation is not as smooth, we further investigated the influence of the different segmentation steps of the semi-automated segmentation.

In the automated segmentation, each step introduced certain rules in order to determine if voxels belong to the RWN. Applying those rules to the manually segmented RWNs allows us to study the origin of the differences between the manual and semi-automated segmentation. Cropping the implant on the outside (toward the middle ear cavity) removes 16 ± 11% of the voxels (i.e., decreases the volume of the niche), cropping inside the RWN (towards the cochlea) removes 19 ± 7% of the manual segmentation, applying the threshold for bone removes 21 ± 8% of the voxels. When all the rules are applied on the manual segmentation, the remaining segmentations have 7 ± 6% less volume compared to the semi-automated segmentations.

### 3.2. Ex-Vivo Validation: Blinded, Comparative RNI Implantations in Human Cadaver Temporal Bones

Using three temporal bones, the otorhinolaryngologist considered the general handling prior to the insertion of all RNI (manually segmented and semi-automatically segmented) including their handle with the forceps as feasible. Direct visual contact with the tip of the instrument and with the RNI could be sufficiently obtained throughout the handling towards the RWN using a binocular microscope. The handle on the surface of all RNI enabled appropriate handling with the forceps and the arrowed side of the handle provided information about the orientation in which the RNI should be implanted. During visualization of the RWN in one temporal bone obstructions were visible in the RWN which were removed before insertion of the RNI.

After the handling, image documentation and rating of the implantations of both RNI—manually segmented and semi-automatically segmented—in each temporal bone the RNI were unblinded.

**Figure 7:**
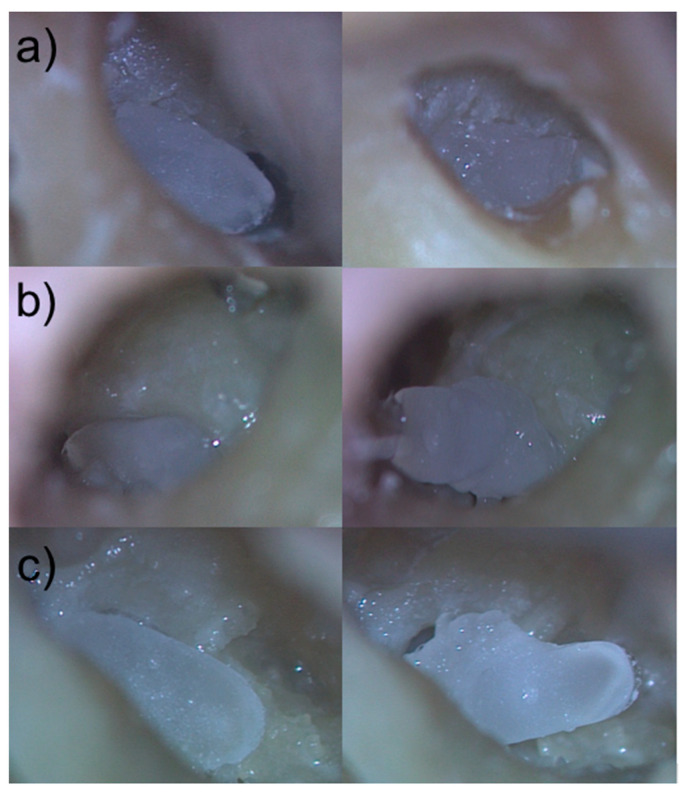
Comparison of manually segmented to semi-automatically segmented and 3D printed RNI in the corresponding human cadaver RWN. (**a**–**c**) left images: 3D printed individualized semi-automatically segmented RNI of the human cadaver RWN in their final position in the RWN (**a**–**c**) right images: 3D printed individualized manually segmented RNI of the human cadaver RWN laying above the bony edges of the RWN.

The manually segmented RNI did not fit into the corresponding RWN in all three temporal bones. The volume of the RWN seemed to be too large to pass the border of the bony edges of the RWN. Several attempts to press the manually segmented RNI into the corresponding RWN failed (Figure 7).

The otorhinolaryngologist rated the fitting of all semi-automatically segmented RNI as good, with all implants sitting pressure-tight in the RWN, allowing visualization of all bony edges of the RWN without room for wobbling in the RWN (Figure 7).

Assembly time for the insertion of the semi-automated segmented RNI was less than ten seconds in all three implantations and the total time from the beginning of the transmeatal approach to final positioning of the RNI in the RWN was less than 10 min. Table 2 depicts the rating matrix for the individual read outs of the ex-vivo validation of the semi-automated and manual segmentation based RNI.

## 4. Discussion

Traditional studies on temporal bones mainly involved cadaveric dissections and histopathologic analysis [29], but with the introduction of new imaging techniques there has been a renewed interest in anatomic analysis [30]. Segmentation of temporal bone structures in 3D is important for surgical planning [31] of otological surgeries or lateral skull base approaches, robotic surgery [32], preoperative surgical training [33], patient-specific cochlear implant programming [34,35] and patient-specific models or implants. Unfortunately, manual segmentation is very labor intensive and not practical in a clinical setting, therefore many groups have been working on automating segmentation with polynomial functions [36], atlas-based registration [22], statistical shape models [37,38,39], and deep learning. These algorithms can then be used to build anatomical models from clinical imaging datasets, allowing accurate 3D reconstruction of a patient’s anatomy [33].

This is the first study to use a semi-automated approach for segmentation of the RWN anatomy. Our group focused on developing this approach since it is an important step in the process of establishing 3D printed individualized implants for RWN based drug delivery to the inner ear. The advantages of our semi-automated approach of RWN segmentation are fourfold: (1) it requires only little manual input, (2) yields segmentation results surpassing those created by trained experts because it avoids over-segmentation as demonstrated in the ex-vivo validation, (3) delivers results considerably faster than manual. (4) Non-expert users can produce better results (i.e., less over-segmentation) with the help of the software than the experienced surgeons performing the manual segmentation. The semi-automated method avoids over-segmentation mainly because it is not influenced by the windowing (brightness/contrast) setting of the DICOM viewer, which makes it more reliable.

Additive manufacturing of drug loaded individualized implants that can be positioned in the individually shaped RWN may overcome the uncontrolled delivery of drugs to the inner ear. Additive manufacturing, also referred to as 3D printing, enables to create implants adjusted to the individual anatomical needs of a patient [40,41,42]. Today, 3D printing technologies already offer many useful applications in the development of new therapies, making 3D printing increasingly important in the healthcare and pharmaceutical industries [43]. 3D technology has already been successfully used for the production of tailor-made prostheses and implants [43]. And the technology holds promising potentials for patient specific drug-loading of 3D printed implants [44]. Furthermore, 3D printed drug delivery devices may lead to more reliable results in future studies on local drug delivery to the inner ear and therefore to a benefit for MD and ISSHL patients.

The first step in developing additively manufactured implants requires images obtained from a comprehensive CT or CBCT scan of the region to be implanted. These images are used to produce a computer-aided design drawing, STL file format respectively, of the object to be manufactured [45]. Even though it is a very time-consuming task, manually segmenting of preoperative images for obtaining a STL file is possible. But it is prone to user variability and inconsistency [21,46] and not feasible for an intraoperative workflow.

Manual segmentation required focused user attention and took ~ 30 min for labeling the RWN, whereas the semi-automated application took only three to five minutes for the development of an RNI. This 8 to 10-fold acceleration of the segmenting process demonstrates the efficiency gains of the semi-automated segmentation approach and facilitates the development of STL files needed to 3D print RNIs for clinical use. This study also presents an efficient semi-automated approach for segmentation of sensitive structures such as RWN and RWM in CBCT scans that is not dependent on the use of a large number of training images. The semi-automated segmentation of the RWN volume of the experienced User 1 was only 13 ± 12% smaller than the segmentation of the RWN volume of User 2. The semi-automated segmentation of the RWM area of User 1 was only 5 ± 17% smaller than the RWM User 2. User 2 was not familiar with segmenting temporal bone structures at all but generated similar results as the trained and experienced User 1 (Figure 6). The finding that by using the semi-automated approach, both user yield consistent segmentation, is supported by the high DSC(Semi_U1_, Semi_U2_) and the J(Semi_U1_, Semi_U2_) (Table 1).

The volume of the manually segmented RWN was 48 ± 11% bigger than the volume of the corresponding RWN using the semi-automated approach and this also explains the relatively small DSC and J between the semi-automated approach and the manual segmentations (Table 1). Cropping variation among temporal bone images from their CT scans can lead to inadequate segmentation of structures at specific anatomical boundaries. There are two difficulties involved for the manual segmentations: One is that the RWN is a half-open structure and the second is that the borders towards bone is somewhat dependent on the windowing settings (contrast/brightness) of the DICOM viewer.

Excluding overlapping areas and removing voxels from the RWN and RWM analysis, as shown in Figure 6, may have produced more accurate segmentations of the RNI. This was also shown in the blinded, comparative human cadaver experiments where different manually and semi-automatically segmented RNI were tested. The human cadaver implantations revealed not only a very good fit of the semi-automatically segmented RNI in the human cadaver RWN but even a pressure-tight fit without room for wobbling in the RWN. The manually segmented RNI did not fit into the corresponding RWN, nor could they pass the bony edges of the RWN to be inserted (Figure 7). Pressuring of the manually segmented RNI into the RWN did not work and carries the risk of damaging the sensitive RWM. Accurate segmentation of surface-based structures is important because they represent key boundaries of the RWN that need to be preserved during insertion of the RNI by the surgeon to avoid injury to deeper structures such as the RWM.

Limitations of our study include that a small number of users tested the semi-automated approach and could make suggestions for suitable iterations and only anatomically normal temporal bones were segmented. There is also a need to investigate how the segmentation algorithms performs in relation to abnormal anatomy of the temporal bone such as vestibulocochlear malformations [21]. In addition, in this study, we used a small sample size of human cadaver temporal bones for the insertion of RNI to test the generalization ability of the proposed model of semi-automated segmentation and fitting of the implants and provided a preliminary research basis for clinical application. Future studies have to investigate how the segmentation algorithms perform in relation to the tissue-implant-interface of the RNI in the RWN and specifically the contact of the RNI to the RWM. Therefore further research, i.e., histopathological cuts of the implanted RNI or post-insertion CT scans, are needed.

While there is much progress in deep learning and related methods for fully automated segmentations, we did not find any literature specifically for the RWN. A prerequisite for deep learning is a large high quality training data set. As it turns out to be a difficult manual task and just training a deep learning network would likely just replicate the human over-segmentations. Therefore, we hope that our semi-automated tool can help to collect enough high quality and human reviewed segmentations to train a deep learning network in the future.

The aim of the study was to write a plug in software for a semi-automated segmentation of the RWN to ease the segmentation of the region for a more efficient workflow of additively manufactured individualized drug delivering RNI. We did not aim to evaluate whether semi-automated segmentation is better or worse than manual segmentation but aimed to validate our approach using manually generated data sets. But in the ex-vivo experiments we figured out that the manually segmented RNI did not fit into the RWN but the RNI based on our written software did nicely fit into the respective individual niche. Therefore, we can state that the developed plug allows a segmentation which is so close to the real anatomical condition that based on this software 3D printed RNI fit precisely into the individual RWN.

## Figures and Tables

**Table 1:** Dice similarity coefficients (DSC) and Jaccard indices (J) between the semi-automatic segmentation Semi_U1_ and Semi_U2_ and the manual reference segmentation Man_U1_. Further, the comparison between the two users U1 and U2 using the semi-automated method is shown. The latter is denoted as DSC(Semi_U1_, Semi_U2_) or J(Semi_U1_, Semi_U2_), respectively.

	Mean	STD
DSC(Semi_U1_, Man_U1_)	0.61	0.08
DSC(Semi_U2_, Man_U1_)	0.55	0.12
DSC(Semi_U2_, Semi_U1_)	0.87	0.09
J(Semi_U1_, Man_U1_)	0.44	0.08
J(Semi_U2_, Man_U1_)	0.39	0.11
J(Semi_U2_, Semi_U1_)	0.78	0.13

**Table 2:** Rating matrix for performance evaluation of the RNI with a rating scale from 1 to 6. 1: very good (green); 2: good (light green); 3: acceptable (yellow); 4: sufficient (light orange); 5: inadequate (orange); 6: insufficient (red).

Criteria	RNI	Rating					
		1	2	3	4	5	6
Handling with forceps	manual	1	2	3	4	5	6
	semi-automated	1	2	3	4	5	6
Time consumption of insertion	manual	1	2	3	4	5	6
	semi-automated	1	2	3	4	5	6
Fitting accuracy	manual	1	2	3	4	5	6
	semi-automated	1	2	3	4	5	6

## Data Availability

The data is presented within the article.
